# 

**Published:** 1801-04

**Authors:** 


### TO CORRESPONDENTS.

*Communications are received from Dr. Adams, Messrs. Headly, Edlin, Swift, Ring, M'Garth, and D. F.*

